# Application of machine learning in prediction of bone cement leakage during single-level thoracolumbar percutaneous vertebroplasty

**DOI:** 10.1186/s12893-023-01959-y

**Published:** 2023-03-23

**Authors:** Guobing Deng, Jichong Zhu, Qing Lu, Chong Liu, Tuo Liang, Jie Jiang, Hao Li, Chenxing Zhou, Shaofeng Wu, Tianyou Chen, Jiarui Chen, Yuanlin Yao, Shian Liao, Chaojie Yu, Shengsheng Huang, Xuhua Sun, Liyi Chen, Zhen Ye, Hao Guo, Wuhua Chen, Wenyong Jiang, Binguang Fan, Zhenwei Yang, Wenfei Gu, Yihan Wang, Xinli Zhan

**Affiliations:** 1 The First Affiliated Hospital of Guangxi Medical University, Nanning, 530021 People’s Republic of China; 2 The First People’s Hospital of Chenzhou, Chenzhou, 423000 People’s Republic of China

**Keywords:** OVCFs, PVP, Machine learning algorithms, Prediction model, Nomogram

## Abstract

**Background:**

In the elderly, osteoporotic vertebral compression fractures (OVCFs) of the thoracolumbar vertebra are common, and percutaneous vertebroplasty (PVP) is a common surgical method after fracture. Machine learning (ML) was used in this study to assist clinicians in preventing bone cement leakage during PVP surgery.

**Methods:**

The clinical data of 374 patients with thoracolumbar OVCFs who underwent single-level PVP at The First People's Hospital of Chenzhou were chosen. It included 150 patients with bone cement leakage and 224 patients without it. We screened the feature variables using four ML methods and used the intersection to generate the prediction model. In addition, predictive models were used in the validation cohort.

**Results:**

The ML method was used to select five factors to create a Nomogram diagnostic model. The nomogram model's AUC was 0.646667, and its C value was 0.647. The calibration curves revealed a consistent relationship between nomogram predictions and actual probabilities. In 91 randomized samples, the AUC of this nomogram model was 0.7555116.

**Conclusion:**

In this study, we invented a prediction model for bone cement leakage in single-segment PVP surgery, which can help doctors in performing better surgery with reduced risk.

## Introduction

In the elderly, osteoporotic vertebral compression fractures (OVCFs) of the thoracolumbar vertebra are common. The incidence of osteoporosis is high in China, with studies estimating that the incidence is around 13% [1]. OVCFs are the most common osteoporosis complication; some metastatic tumors can cause vertebral rupture and compression fractures. Spinal fractures are becoming more common each year, affecting the quality of life of older adults. These OVCFs lead to short-term and long-term back pain, severe vertebral deformity, and functional disability, which seriously affect the quality of life in elderly patients [2]. Various treatments for OVCFs have been developed for more than 100 years. Anesthesia and internal fixation techniques have recently gained popularity, but there is still great controversy regarding the best treatment plan for OVCFs [3].

Percutaneous vertebroplasty (PVP) is a minimally invasive technique in which liquid polymethylmethacrylate, known as bone cement, is injected into a fractured vertebral body through a hollow puncture needle to strengthen and prevent further compression of the vertebral body [4]. PVP is frequently used to treat OVCFs and spinal tumors [5]. The penetration direction of bone cement in the vertebral body is different due to the presence of multiple vertebral bone destruction. Extravasation of bone cement into the epidural or intervertebral foramen compresses the spinal cord and nerve roots, increasing the risk of adjacent vertebral body fracture and even resulting in paraplegia [6]. In addition, the extravasation of bone cement into paraspinal veins can pose serious complications like pulmonary embolism, cardiac perforation, cerebral embolism, and even death [7]. Cement leakage often occurs in PVP, but clinicians lack a good method to predict the probability of cement leakage.

Machine learning (ML) is a new discipline that has emerged in the past 20 years involving many subjects such as probability theory, statistics, approximation theory, convex analysis, and algorithm complexity theory. The machine learning algorithm obtains rules automatically from data analysis and uses these rules to predict unknown data. Machine learning is widely used in clinical data-related research [8, 9].

Our team gathered clinical data from single-stage PVP surgery in OVCF patients in the hopes of screening out patients with bone cement leakage using four machine-learning methods and developing a prediction model. This model may aid clinicians in preventing bone cement leakage during PVP surgery.

## Patients and methods

All the subjects volunteering for this study signed the informed consent forms. The study was approved by the Ethics Committee of The First People’s Hospital of Chenzhou.

From 2020 to 2022, 374 patients with OVCFs who underwent single-segment thoracolumbar PVP at the First People's Hospital of Chenzhou were chosen. There were 150 patients with bone cement leakage and 224 patients without. The following variables were selected for the analysis: age, sex, BMI, associated with old vertebral fractures, previous PVP operation history, combined with thoracolumbar kyphosis, heart or lung disease, classification of fracture, degree of vertebral compression, preoperative MRI showing signal changes in the injured vertebrae, time of spinal fracture, history of trauma, lumbar vertebra bone density examination, surgical vertebral segment, puncture path, VAS score, VAS score 24 h after surgery, VAS score at discharge, bone cement injection volume, pre and post-penetration of bone cement, and upper and lower penetration of bone cement. In the end, 91 patients were randomly sampled in the experimental group for internal validation. Old fractures were defined as patients having fractures longer than 3 weeks in a single segment [10]. The preoperative MRI showing signal changes in the injured vertebrae was collected, and the adjacent vertebrae served as a reference to locate the MRI signal changes in the fractured vertebrae.

All clinical data were obtained from the information system of The First People's Hospital of Chenzhou, and other data were obtained from the questionnaires given to the patients at the time of admission and the telephonic follow-up after surgery. Inclusion criteria were: (1) patient had a thoracolumbar fracture and was diagnosed with osteoporosis by the doctor; (2) chest, waist, and back pain, limited activity affecting the quality of life, and conservative treatment is not effective; (3) fresh or relatively fresh osteoporotic compression fractures diagnosed with imaging; (4) the overall bone mineral density examination was normal, local vertebral metastases caused vertebral osteoporosis, or the clinician diagnosed vertebral osteoporosis in the patient based on clinical manifestations and X-ray examination, and the patient underwent PVP surgery; (5) acceptable compliance and tolerance of patients having no absolute contraindications such as serious cardiovascular and cerebrovascular diseases. Exclusion criteria were: (1) in patients with normal bone, trauma resulted in thoracolumbar fractures; (2) case fracture caused by systemic or local infection; (3) severe compression fracture, secondary spinal canal stenosis, spinal instability, or symptoms of nerve compression requiring decompression and fixation surgery; (4) PVP performed in multiple vertebrae concurrently; (5) patients with intolerance or coagulopathy, as well as other surgical contraindications.

### Statistical analysis

For data analysis, we used IBM SPSS Statistics 23. The student t-test is used to compare continuous variables with normal distributions of variance homogeneity. The chi-square test was used to analyze patient data composition ratio and frequency distribution, and SPSS creates different variables. A probability of < 0.05 on both sides was considered statistically significant for all analyses.

All statistical analyses were performed using R software (version 4.1.3; https://www.R-project.org). The nomogram survival model was constructed to predict cement leakage in PVP surgery by using R software’s “rms” and “regplot” package [11]. The “rms” package was also used to calculate the C value and univariate logistic regression to verify the nomogram model’s prediction ability [12, 13]. The AUC (area under the curve) of the ROC curve and Harrell’s concordance index was used to evaluate the performance of nomogram predictions. Harrell’s concordance index was calculated to quantify nomogram discrimination by using a bootstrap method with 1000 samples [14]. Decision curve analysis was conducted to determine the clinical usefulness of the non-adherence nomogram by quantifying the net benefits at different threshold probabilities in patients with cement leakage after PVP surgery [15]. The thresholds were obtained and visualized using the “rms” and “rmda” packages.

### Machine learning

#### Univariate logistic regression

Univariate logistic regression was used to investigate the independent risk factors for bone cement leakage after PVP. The 95% confidence interval (CI) and odds ratio (OR) were calculated by the backward stepwise selection method. OR > 1 indicates that the variable is a risk factor, and *P* < 0.05 was considered statistically significant [16].

#### LASSO regression

Least absolute shrinkage and selection operator is the full name of LASSO regression. This method is a compression estimation method that uses general linear regression to add regular terms. The parameters are kept as simple as possible while ensuring the best-fitting error, and the model has strong generalization ability. The LASSO regression model actively selects from a large multi-collinear set of potential variables in the regression analysis to screen out risk factors, and best predict cement lateral leakage characteristics from PVP data [17]. We used the "glmnet" package in R software for LASSO regression analysis and visualization [18].

#### Random forest

Random forest is a compositional supervised machine learning algorithm. In this study, we used the "randomForest" package in R software to screen out variables, calculate their relative importance, and visualize them [19]. The randomForest package includes two screening methods: an increase in mean squared (%IncMSE) and an increase in node purity (IncNodePurity). The relationship between model errors and fitted variables is depicted by cross-validation curves. Multiple groups of different training and verification are performed on the model in this study by using different training sets and verification set division through the implementation of ten-fold cross-validation, to deal with the problems of one-sided test results and insufficient data [20].

#### SVM-RFE

In SVM-RFE (Support Vector Machine-Recursive Feature Elimination), the required variables were screened out and visualized using the "e071" package in R software. tenfold cross-validation was carried out for the data, and a characteristic index of the vector was obtained. The smaller the AvgRank number, the greater the influence of this factor on bone cement leakage in PVP. After sorting, we estimated the generalization error for all data and finally screened the variable with the lowest common diagnosis error rate [21].

## Results

Table 1 shows all the variables collected for machine learning. The proportion of female patients with thoracolumbar fractures was higher than males, and there was no statistical difference between gender and bone cement leakage. The rate of osteoporosis in female patients was found to be significantly high than that in male patients (Table 2). The percentage of bone cement leakage in patients with old spinal fractures was significantly lower than that in patients with new fractures. As reported in Table 1, 68 patients had PVP before, and the proportion of bone cement leakage in patients with no previous history of PVP increased. Cement leakage was found more common in patients without thoracolumbar kyphosis. As shown in Table 3 of thoracolumbar kyphosis, we found in training cohort no statistical difference in the amount of intraoperative bone cement injection in patients with thoracolumbar kyphosis. Patients with severe splitting, pathologic, and vertebral compression fractures were more likely to have cement leakage, but we observed no statistical difference. Traumatic thoracolumbar fracture is a fracture of the thoracolumbar spine caused by trauma in osteoporosis patients. Fracture of the vertebral body caused by trauma is prone to cement leakage. Leakage rates were observed to be higher in patients without osteoporosis and osteopenia than in patients with osteoporosis. Patients who have experienced trauma are more likely to experience bone cement leakage. There was no statistically significant difference in the incidence of leakage following various surgical procedures.

**Table 1:** Baseline table of patients with and without cement leakage

**Type**	**Yes** (*N* = 150)	**NO** (*N* = 224)	***P***	**Type**	**Yes** (*N* = 150)	**NO** (*N* = 224)	***P***
**Age**				**Lumbar vertebra bone density examination**			
Mean(SD)	69.47 (9.47)	71.55 (9.89)	0.42	Normal (*n* = 21)	11 (52.4%)	10 (47.9%)	**0.006**
**BMI**				Osteopenia (*n* = 71)	39 (54.9%)	32 (45.1%)	
Mean(SD)	22.57 (2.68)	22.32 (2.81)		Osteoporosis (*n* = 282)	100 (35.5%)	182 (64.5%)	
**Bone cement injection volume**			0.374	**Surgical vertebral segment**			
Mean(SD)	4.57 (0.89)	4.61 (0.86)		Thoracic vertebra (*n* = 218)	92 (42.2%)	126 (57.8%)	0.601
**Sex**				Lumbar vertebra (*n* = 147)	58 (39.5%)	89 (60.5%)	
Male	37	57	0.657	**Puncture path**			
Female	113	167		Conventional puncture route (*n* = 299)	125 (41.8%)	174 (58.2%)	0.181
**Associated with old vertebral fractures**				Lateral puncture path (*n* = 75)	25 (33.3%)	50 (66.7%)	
Yes (*n* = 138)	46 (33.3%)	92 (66.7%)	**0.046**	**VAS score**			
No (*n* = 235)	103 (43.8%)	132 (56.2%)		No	0 (	0	0.903
**Previous PVP operation history**				1–3	0	0	
Yes (*n* = 68)	20 (29.4%)	48 (70.6%)	**0.047**	4–6 (*n* = 156)	62 (39.7%)	94 (60.3%)	
No (*n* = 306)	130 (42.5%)	176 (57.5%)		7–10 (*n* = 218)	88 (40.4%)	130 (59.6%)	
**Combined with thoracolumbar kyphosis**				**VAS score 24 h after surgery**			
Yes (*n* = 85)	21(24.7%)	64 (75.3%)	**0.01**	No	0	0	0.243
No (*n* = 289)	129 (44.6%)	160 (55.4%)		1–3 (*n* = 371)	149 (40.2%)	222 (59.8%)	
**Heart or lung disease**				4–6	0	2	
Yes (*n* = 58)	19 (32.8%)	39 (67.2%)	0.214	7–10	1 (0.7%)	0 (0%)	
No (*n* = 316)	131 (41.5%)	185 (58.4%)		**VAS score at discharge**			
**Classification of fracture**				No (*n* = 48)	23 (47.9%)	25 (52.1%)	0.237
Simple compression fracture (*n* = 355)	140 (39.4%)	215 (60.6%)	0.516	1–3 (*n* = 326)	127 (39%)	199 (61%)	
Cleavage fracture (*n* = 15)	8 (53.3%)	7 (46.7%)		4–6	0	0	
Pathological fracture (*n* = 4)	2 (50%)	2 (50%)		7–10	0	0	
**Degree of vertebral compression**				**Pre and post penetration of bone cement**			
< 25% (*n* = 218)	78 (35.8%)	140 (64.2%)	0.120	Bone cement did not cross the midline (*n* = 22)	12 (54.5%)	10 (45.5%)	0.186
26–40% (*n* = 85)	38 (44.7%)	47 (55.3%)		The bone cement did not cross the midline and did not cross the medial projection of the pedicle (*n* = 100)	44 (44%)	56 (56%)	
> 40% (*n* = 71)	34 (47.9%)	37 (52.1%)		The bone cement crosses the midline beyond the medial projection of the pedicle (*n* = 252)	94 (37.3%)	158 (62.7%)	
**Preoperative MRI showed signal changes in the injured vertebrae**				**Upper and lower penetration of bone cement**			
Middle and upper vertebral body (*n* = 116)	48 (41.4%)	68 (58.6%)	0.935	The bone cement is not connected to the upper and lower end plates (*n* = 321)	130 (40.5%)	191 (59.5%)	0.519
Middle and lower vertebral body (*n* = 17)	7 (41.2%)	10 (58.8%)		Bone cement is not connected to upper and lower end plates (*n* = 3)	1 (33.3%)	2 (66.7%)	
The upper and lower parts of the vertebral body (*n* = 241)	95 (39.4%)	146 (60.6%)		The bone cement was not attached to the lower endplate (*n* = 35)	11 (31.4%)	24 (68.6%)	
**History of trauma**				The bone cement was not attached to the end plate (*n* = 15)	8 (53.3%)	7 (46.7%)	
Yes (*n* = 171)	81 (47.4%)	90 (52.6%)	**0.009**				
No (*n* = 203)	69 (34%)	134 (66%)					

*VAS* Visual Analogue Score, *P P*-value

**Table 2:** Bone mineral density between male and female patients

Type	Normal	Osteopenia	Osteoporosis	*P*-value
Male (*n* = 57)	5 (8.8%)	18 (31.6%)	34 (59.6%)	** < 0.001**
Female (*n* = 167)	5 (3%)	15 (9%)	147 (88%)

**Table 3:** Relationship between thoracolumbar kyphosis and intraoperative bone cement injection

	**Yes (*N* = 85)**	**No (*N* = 285)**	***P*-value**
**Bone cement injection volume**			
Mean(SD)	4.55(0.92)	4.60(0.86)	0.644
Median【Min, Max】	4.5【3, 8.5】	4.8【2, 7.5】	

Yes: Combined with thoracolumbar kyphosis. No: No combined with thoracolumbar kyphosis

**Fig. 1:**
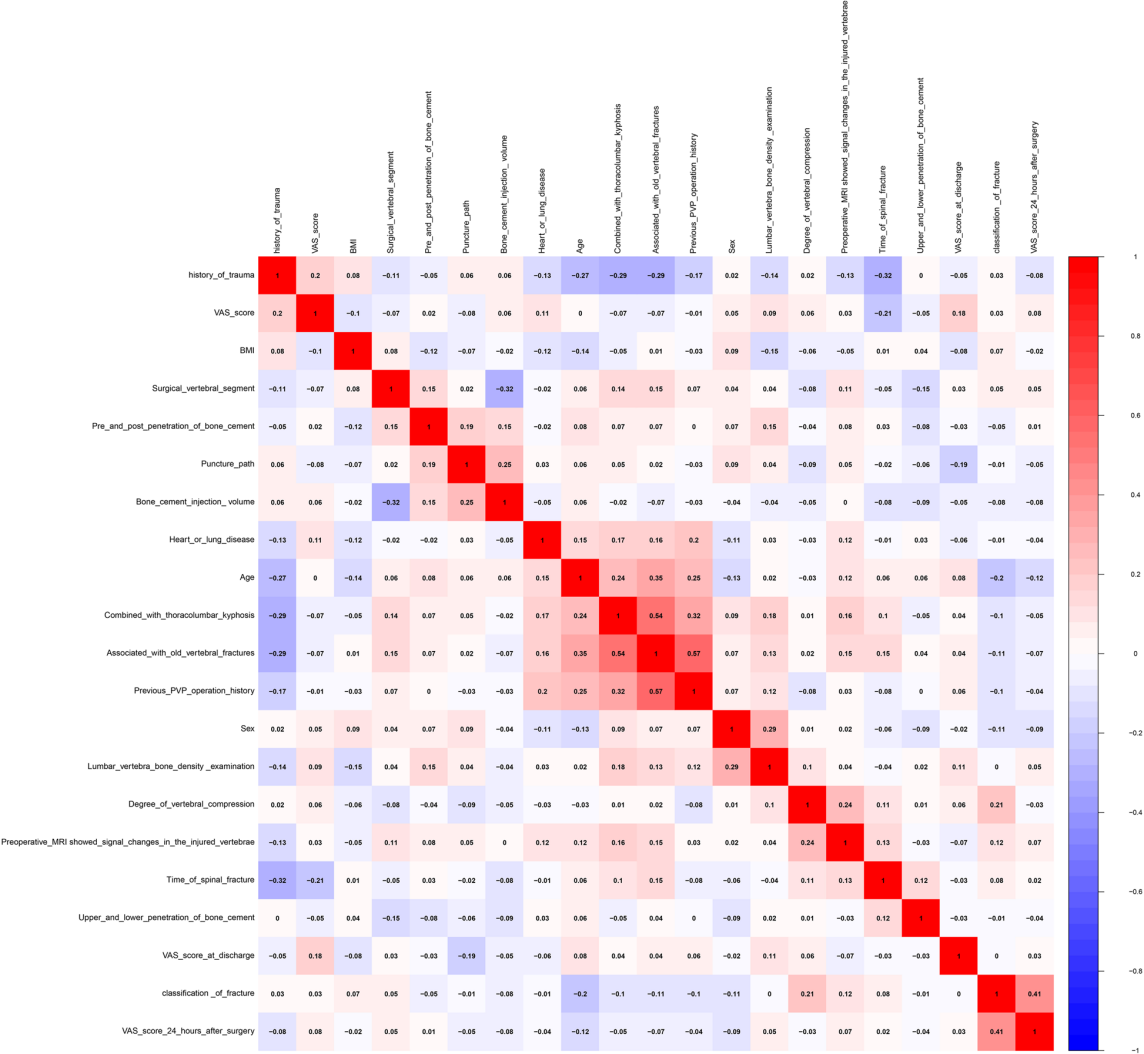
Heat map of the correlations between all the variables are shown

In the correlation diagram of variables shown in Fig. 1, the variable-associated with old vertebral fractures was found to be positively correlated with two other variables-combined with thoracolumbar kyphosis and previous PVP operation history. Other variables were not found to correlate closely.

### Machine learning

#### Univariate logistic regression

Single-factor logistic regression results are shown in Table 4. Various variables such as age, associated with old vertebral fractures, previous PVP operation history, combined with thoracolumbar kyphosis, degree of vertebral compression, history of trauma, and lumbar vertebra bone density examination were found to be statistically different.

**Table 4:** Univariate logistic regression results

Type	OR(95% CI)	*P*-value
Age	0.9778 (0.9566–0.9991)	**0.0425**
Sex	1.0424 (0.6485–1.6895)	0.8647
BMI	1.0348 (0.9599–1.1161)	0.3730
Associated with old vertebral fractures	0.6346 (0.4078–0.9794)	**0.0416**
Previous PVP operation history	0.5641 (0.3136–0.9833)	**0.0485**
Combined with thoracolumbar kyphosis	0.4070 (0.2316–0.6916)	**0.0012**
Heart or lung disease	0.6880 (0.3738–1.2294)	0.2159
Classification. of fracture	1.4633 (0.7044–3.1187)	0.3045
Degree of vertebral compression	1.3058 (1.0053–1.6980)	**0.0456**
Preoperative MRI showed signal changes in the injured vertebrae	0.9526 (0.7623–1.1923)	0.6698
Time of spinal fracture	0.9309 (0.7033–1.2255)	0.6125
History of trauma	1.7478 (1.1526–2.6598)	**0.0088**
Lumbar vertebra bone density examination	0.5873 (0.4051–0.8433)	**0.0043**
Surgical vertebral segment	0.8106 (0.5305–1.2337)	0.3288
Puncture path	0.6960 (0.4038–1.1753)	0.1820
VAS score	1.0263 (0.6750–1.5637)	0.9035
VAS score 24 h after surgery	0.8631 (0.1856–2.8007)	0.8107
VAS score at discharge	0.6937 (0.3770–1.2809)	0.2388
Bone cement injection volume	0.9472 (0.7443–1.2015)	0.6556
Pre and post penetration of bone cement	0.7280 (0.5147–1.0275)	0.0709
Upper and lower penetration of bone cement	0.9944(0.7637–1.2836)	0.9658

#### LASSO regression

**Fig. 2:**
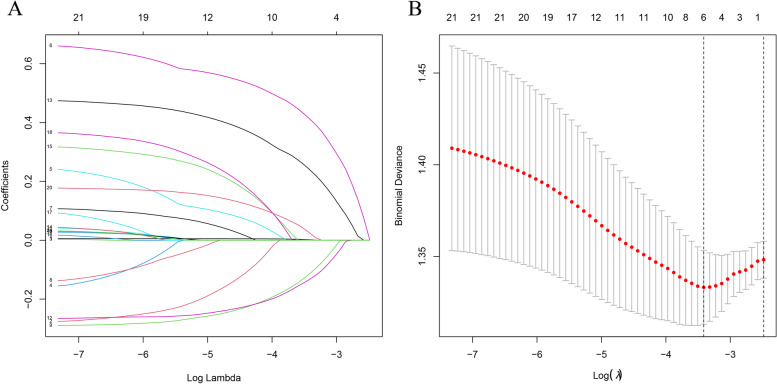
Using cross-validation to the optimal penalty parameter lambda, LASSO coefficient profiles of the factors. **A** The results of the LASSO regression analysis of dependent variables. **B** The 6 factors that exhibited significant differences between the patients with HLA-B27 positive and negative patients

Figure 2A depicts a partial plot of LASSO regression coefficients, with different colored lines representing different variables. Figure 2B cross-validates the curve and reveals six significant differences in patients with bone cement leakage, including age, thoracolumbar kyphosis, degree of vertebral compression, history of trauma, lumbar vertebra bone density examination, and pre and post-penetration of bone cement.

#### Random forest

**Fig. 3:**
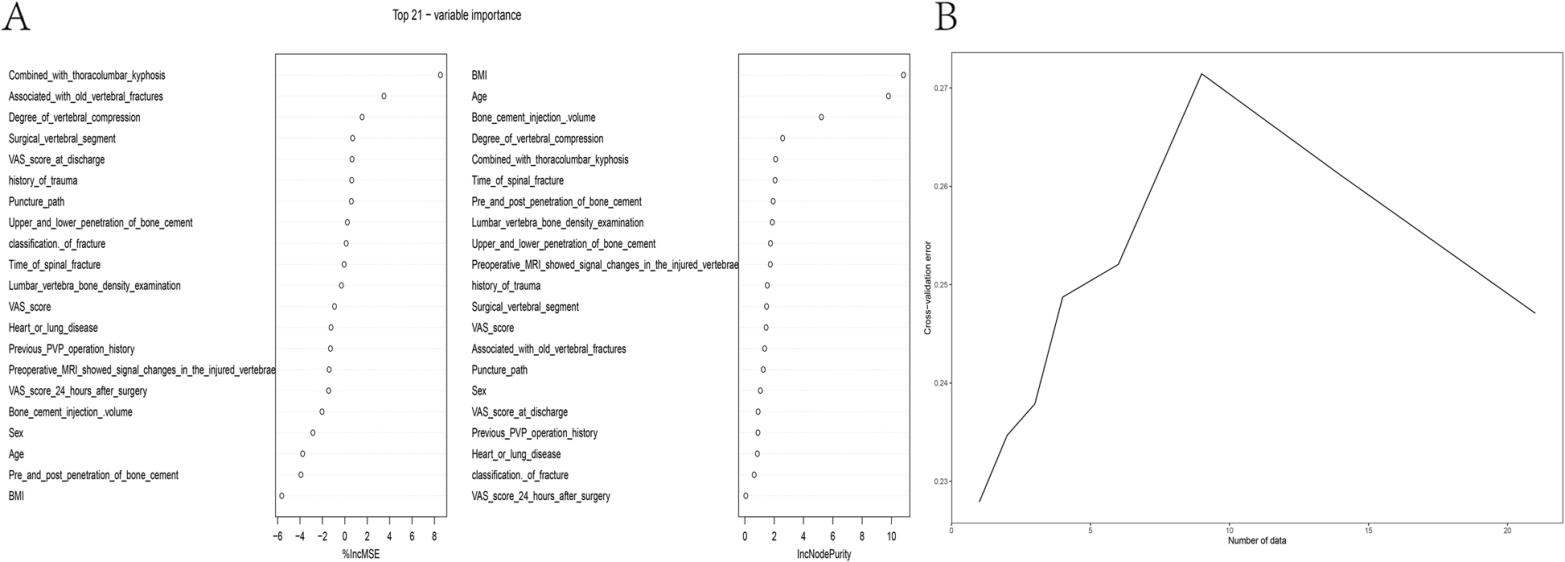
Randomforest screening variables. **A** The 21 most important factors calculated using the two random forest algorithms “%IncMSE” and “IncNodePurity.” **B** the use of the random forest selection model gradually increased the error when 1–10 variables were selected, and then the diagnostic error decreased gradually

Figure 3a shows the ranking of variable importance screened by two random forest methods of "%IncMSE" and "IncNodePurity". Figure 3b shows that the use of the random forest selection model gradually increased the error when 1–10 variables were selected, and then the diagnostic error decreased gradually. Figure 3b shows that random forest is not an ideal prediction method in this prediction.

#### SVM-RFE

**Fig. 4:**
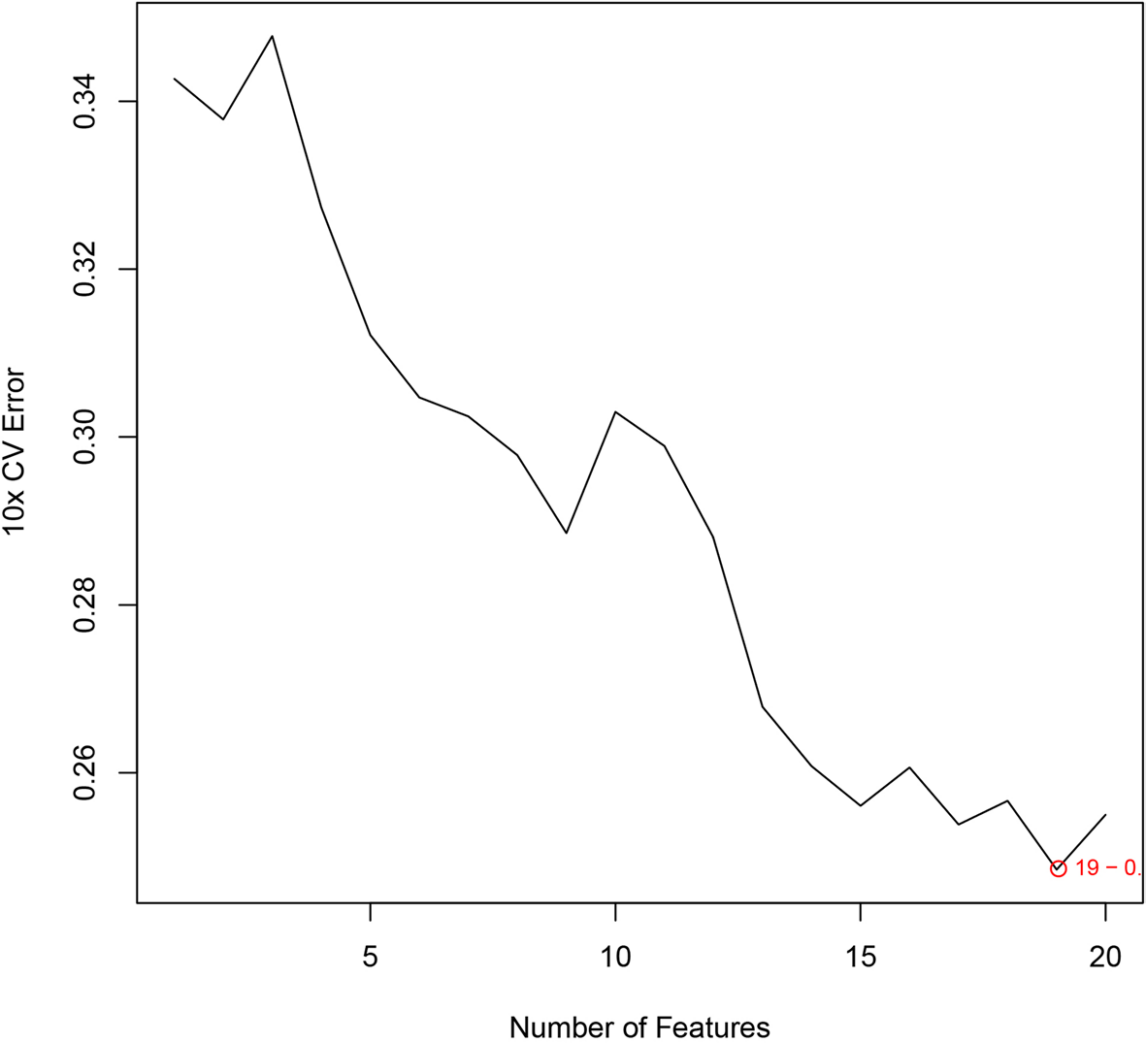
The 19 factors were selected as diagnostic models after SVM-RFE calculation

Figure 4 shows that the error rate reached the minimum when 19 factors were selected as diagnostic models after SVM-RFE calculation. All the included factors were found meaningful for the diagnosis. Table 5 shows the order of importance of 19 factors in the SVM-RFE ranking, and we observed that the importance of a factor increased with a decrease in its AvgRank.

**Table 5:** Importance ranking of 19 factors in SVM-RFE

Type	AvgRank	Type	AvgRank
Lumbar vertebra bone density examination	2.6	**Age**	12.0
Combined with thoracolumbar kyphosis	3.1	**Preoperative MRI showed signal changes in the injured vertebrae**	12.4
Degree of vertebral compression	3.6	**Upper and lower penetration of bone cement**	12.6
Sex	5.9	**Time of spinal fracture**	12.8
Puncture path	6.0	**Heart or lung disease**	13.0
Pre and post penetration of bone cement	9.1	**Surgical vertebral segment**	14.5
History of trauma	9.2	**Bone cement injection volume**	15.2
Associated with old vertebral fractures	9.6	**classification. of fracture**	16.2
VAS score at discharge	10.3	**VAS score**	16.2
Previous PVP operation history	10.8		

**Fig. 5:**
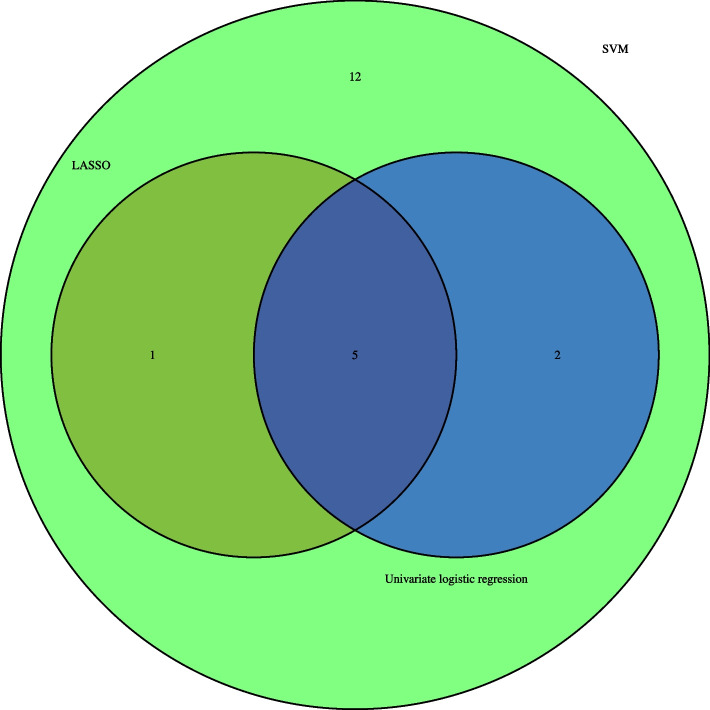
The intersection of variables screened using LASSO, Univariate logistic regression and SVM-RFE

**Fig. 6:**
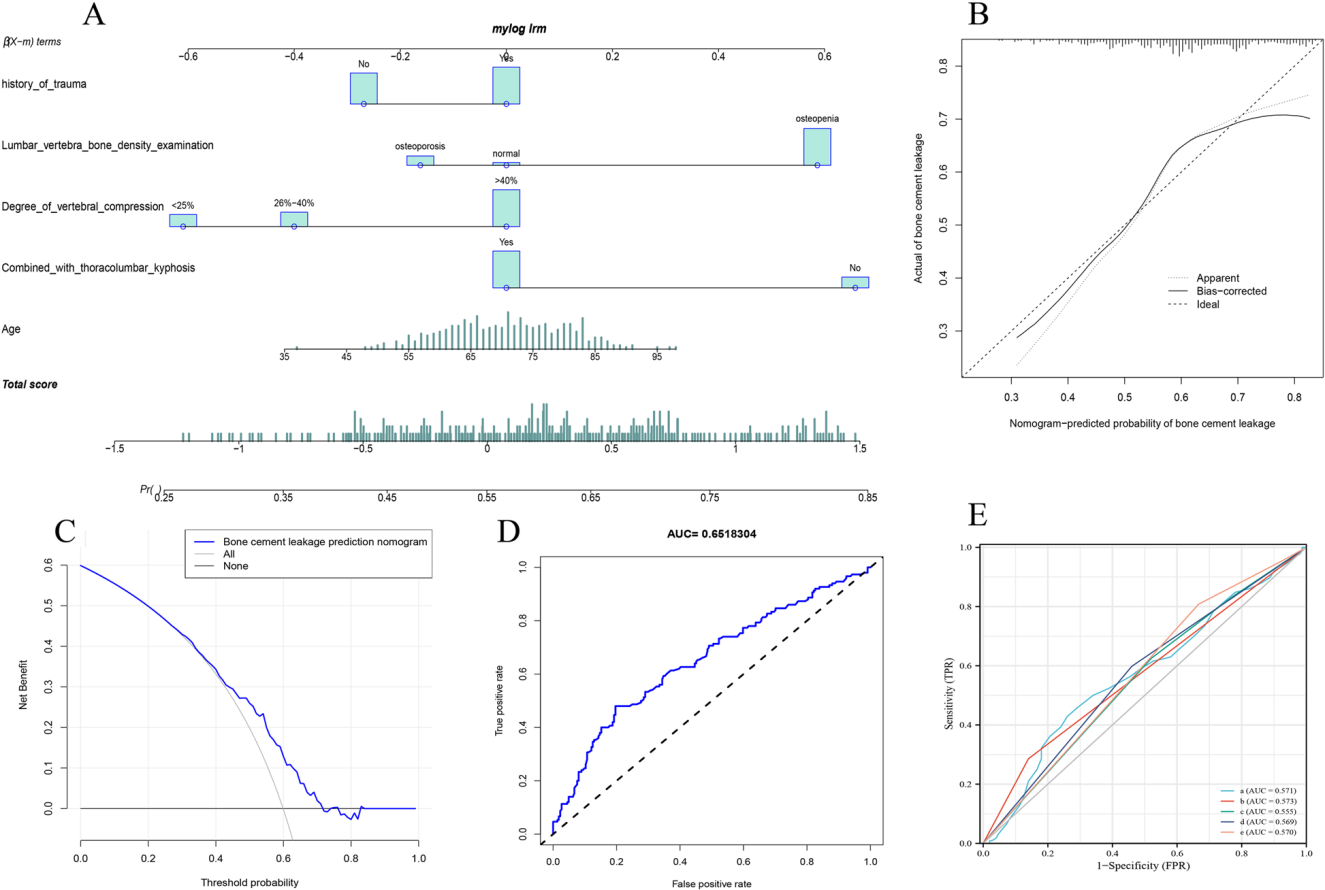
The Prediction model of bone cement leakage diagnostic model. **(A)** Nomogram predicts cement leakage in single-level PVP surgery. **(B)** Calibration curves for predicting cement leakage in single-level PVP surgery. **(C)** Decision curve analysis for the nomogram. **(D)** AUC of the nomogram based on the diagnostic model. **(E)** Individual AUC values of the five variables in the nomogram.** a**, Age. **b**, Combined with thoracolumbar kyphosis. **c**, Degree of vertebral compression. **d,** history of trauma. **e**, Lumbar vertebra bone density examination

We used the intersection of the results of the other three machine learning models (Fig. 5) to obtain five important factors because random forest was not an excellent prediction model in this study. These were age, combined with thoracolumbar kyphosis, degree of vertebral compression, history of trauma and lumbar vertebra bone density examination. Figure 6E shows the AUC values of each factor.

We constructed a nomogram model using these four factors (Fig. 6A). The AUC value of the nomogram model was 0.651834 (Fig. 6D), and the C value was 0.652. The calibration curves showed a reliable agreement between nomogram predictions and actual probabilities (Fig. 6B). The decision curve in Fig. 6C shows that when the threshold value of the model was set in the range of 28%-73%, the curve went above the “none” and “all” lines, and the model showed clinical usefulness in this range.

**Fig. 7:**
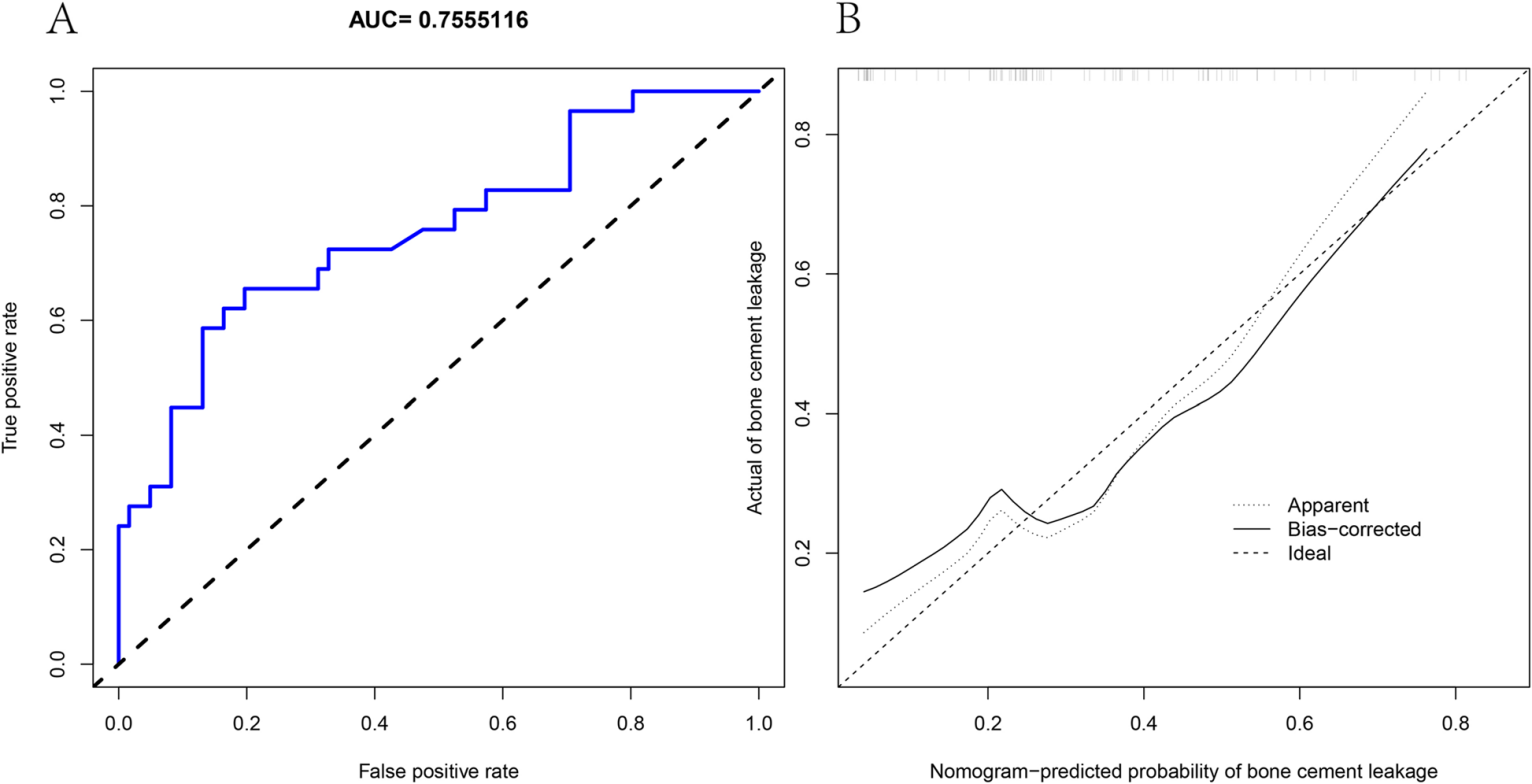
Validation cohort. **a** The AUC value of nomogram diagnostic model in the validation cohort.** b** In validation cohort Calibration curves for nomogram diagnostic model in the validation cohort

An internal random sample of 91 patients was selected for the validation, including 29 patients with postoperative leakage and 61 patients without leakage in PVP. The AUC value of the nomogram model was calculated to be 0.7555116 (Fig. 7A), and the C value was 0.756. The calibration curve showed a satisfactory agreement between nomogram prediction and actual probabilities (Fig. 7B).

## Discussion

In this study, we used clinical data from OVCFs patients who had PVP to create a prediction model for cement leakage after single-vertebra PVP using an ML algorithm. The prediction model was based on a series of predictions and four machine learning models that were used to filter variables, which were further verified by a randomly sampled validation group. By comparing the patients to each other, we learned about the clinical characteristics of patients with leakage in PVP. ML can process clinical data efficiently and optimize model structure by using the intersection method to make the model more concise [22]. This AI-based strategy could be used by clinicians as a preoperative prediction of leakage in patients of OVCFs undergoing PVP surgery.

A higher proportion of females with a thoracolumbar vertebral fracture is associated with osteoporosis [23]. Primary osteoporosis mainly includes senile osteoporosis and postmenopausal osteoporosis. Postmenopausal osteoporosis results in higher bone loss than bone formation due to the massive drop of estrogen in the body. We need to strengthen the publicity and education about the need for calcium supplements during menopause to prevent osteoporosis. Through the machine learning process, we found that the risk of bone cement leakage increases with an increase in age [21]. As patients age, the degree of osteoporosis increases, and along with it, the risk of cement leakage [24].

Thoracolumbar kyphosis may occur as a result of vertebral fractures or age-related spinal degeneration [25]. Severe kyphosis may result in symptoms of nerve or spinal cord compression. PVP surgery can treat kyphosis to some extent [26]. Some researchers believe that thoracolumbar kyphosis is a risk factor for bone cement leakage in PVP. In our study, patients with thoracolumbar kyphosis exhibited a low percentage of bone cement leakage. Thoracolumbar kyphosis might increase the difficulty of puncture to some extent, but we found no significant difference in the amount of injected bone cement in patients with thoracolumbar kyphosis and patients without kyphosis. Reasons specific to the decrease in the percentage of bone cement leakage need further exploration.

Patients with primary osteoporosis or vertebral metastases, as well as obvious post-traumatic thoracolumbar compression fractures, are more likely to have bone cement leakage, which may be related to vertebral fracture morphology. There is a significant difference in the degree of traumatic spinal fracture and simple osteoporotic spinal fracture, with the risk of complications being higher in the case of traumatic fracture [27]. This reminds us that patients with osteoporosis should avoid strenuous activities and avoid trauma, which can lead to OVCFs and bone cement leakage during PVP.

There are very few clinical studies on vertebral compression and bone cement leakage. However, our study found that the more severe the vertebral compression, the greater the possibility of cement leakage in PVP surgery. This might be related to the decrease in the volume of the vertebral body after compression. The smaller the compression volume of the vertebral body, the smaller the space of bone cement, and the more difficult the puncture, and thus, more chances of bone cement leakage [28]. It suggests that clinicians should be more careful when performing PVP in patients with severe vertebral compression and should pay attention to the adjustment of puncture angle and bone cement viscosity. Preoperative MRI showed that the different signal changes of the injured vertebrae did not affect the likelihood of bone cement leakage.

Machine learning models such as univariate logistic regression, LASSO regression, random forest, and SVM-RFE are frequently used to establish various models. However, the performance of various machine learning methods varies. This time, we employ the Venn diagram intersection method to optimize our diagnostic model and the factors derived from various ML methods.

This study aimed to investigate the risk factors for bone cement leakage and to select the best machine learning prediction using a patient dataset containing 374 PVP procedures. Our study has several advantages: (1) we collected a large number of clinical data of PVP patients for the research; (2) we used four machine learning methods to filter the data and use the verification group for validation; (3) our model has a good predictive ability to screen out the high-risk features of bone cement leakage, making it easier for clinicians to perform PVP in OVCF patients.

However, there are some limitations to this study as well: (1) the retrospective studies may lead to subjective and selection bias; (2) our patient data is limited to one hospital, which may limit the use of our prediction model in other areas and require further validation; (3) the predictive performance is unsatisfactory.

## Conclusion

Finally, the prediction model of bone cement leakage after single-level PVP established in this study has good performance, high accuracy, and is easy to use. The prediction model can accurately predict the likelihood of bone cement leakage in OVCF patients undergoing single-level PVP and assist clinicians in better preventing it. We acknowledge, however, that clinicians always have the final say in disease interpretation due to their domain expertise. We hope that our diagnostic model will soon be able to cover a wide range of clinical variables, allowing it to be used accurately in a larger population.

## Data Availability

The original contributions presented in the study are included in the article/supplementary material. Further inquiries can be directed to the corresponding authors.
